# Mfd Affects Global Transcription and the Physiology of Stressed *Bacillus subtilis* Cells

**DOI:** 10.3389/fmicb.2021.625705

**Published:** 2021-01-28

**Authors:** Holly Anne Martin, Anitha Sundararajan, Tatiana S. Ermi, Robert Heron, Jason Gonzales, Kaiden Lee, Diana Anguiano-Mendez, Faye Schilkey, Mario Pedraza-Reyes, Eduardo A. Robleto

**Affiliations:** ^1^School of Life Sciences, University of Nevada, Las Vegas, Las Vegas, NV, United States; ^2^National Center for Genome Resources, Santa Fe, NM, United States; ^3^West Career and Technical Academy, Las Vegas, NV, United States; ^4^The College of Idaho, Caldwell, ID, United States; ^5^Division of Natural and Exact Sciences, Department of Biology, University of Guanajuato, Guanajuato, Mexico

**Keywords:** oxidative stress, protein oxidation, stationary-phase, transcription-coupled repair, *Bacillus*

## Abstract

For several decades, Mfd has been studied as the bacterial transcription-coupled repair factor. However, recent observations indicate that this factor influences cell functions beyond DNA repair. Our lab recently described a role for Mfd in disulfide stress that was independent of its function in nucleotide excision repair and base excision repair. Because reports showed that Mfd influenced transcription of single genes, we investigated the global differences in transcription in wild-type and *mfd* mutant growth-limited cells in the presence and absence of diamide. Surprisingly, we found 1,997 genes differentially expressed in Mfd^–^ cells in the absence of diamide. Using gene knockouts, we investigated the effect of genetic interactions between Mfd and the genes in its regulon on the response to disulfide stress. Interestingly, we found that Mfd interactions were complex and identified additive, epistatic, and suppressor effects in the response to disulfide stress. Pathway enrichment analysis of our RNASeq assay indicated that major biological functions, including translation, endospore formation, pyrimidine metabolism, and motility, were affected by the loss of Mfd. Further, our RNASeq findings correlated with phenotypic changes in growth in minimal media, motility, and sensitivity to antibiotics that target the cell envelope, transcription, and DNA replication. Our results suggest that Mfd has profound effects on the modulation of the transcriptome and on bacterial physiology, particularly in cells experiencing nutritional and oxidative stress.

## Introduction

Short for Mutation frequency decline, Mfd is the transcription-coupling repair factor, which coalesces a stalled RNAP with the Nucleotide Excision Repair (NER) pathway to preferentially repair lesions in the template strand of actively transcribed genes before lesions in the coding strand or in non-actively transcribed genes (Hanawalt and Spivak, 2008). However, as early as the 1990s, evidence suggested that Mfd influenced phenotypes unrelated to transcription-coupled repair. Reports showed that Mfd affected carbon catabolite repression of operons (Zalieckas et al., 1998b). In addition, *in vitro* studies showed that Mfd can facilitate repression of transcription by roadblock clearance at genes regulated by the global transcription regulator CodY (Belitsky and Sonenshein, 2011). Our lab demonstrated a role for Mfd in the expression of amino acid biosynthesis genes and protection from oxidative stress (Pybus et al., 2010; Martin et al., 2011, 2019). Of note, a recent report proposed that Mfd functions at hard-to-transcribe genes and affected gene expression and survival associated with toxin-antitoxin gene modules in *B. subtilis* (Ragheb et al., 2021).

In bacterial species other than *B. subtilis*, observations suggest that Mfd influences traits other than DNA repair. While in some organisms Mfd has the potential to increase the frequency of mutations conferring resistance to antibiotics (Han et al., 2008; Ragheb et al., 2019; Merrikh and Kohli, 2020), in other bacteria such as *H. pylori*, it can increase antibiotic sensitivity (Lee et al., 2009). In *Staphylococcus aureus*, inactivation of *mfd* resulted in decreased biofilm formation (Tu Quoc et al., 2007). Interestingly, *E. coli* cells can perform transcription-coupled repair through an Mfd-independent mechanism and the activity of UvrD (Epshtein et al., 2014). This Mfd-independent transcription-coupled repair has been postulated to occur in *B. subtilis* (Moreno-Del Alamo et al., 2020). Also, DNA lesions of oxidative nature modulate growth and stationary-phase associated Mfd-dependent mutagenic events in this Gram-positive microorganism (Leyva-Sánchez et al., 2020). Moreover, recent single-molecule resolution experiments showed that Mfd can translocate on undamaged DNA independently of its interactions with RNAP (Ho et al., 2018). Given the previous biochemical observations, and the different phenotypes in different bacterial species associated with Mfd, we hypothesized that Mfd, in addition to mediating transcription-coupled repair, modulates the cell transcriptome. More specifically, we tested whether Mfd affects the global transcription profile in stationary-phase *B. subtilis* cells and in conditions of diamide exposure. Diamide is an oxidizing agent that transforms protein thiol groups into disulfide bonds and subject cells to protein oxidation stress (Pother et al., 2009). Cells experiencing disulfide stress activate the oxidative and electrophile stress stimulon to repair and process cellular damage (Antelmann and Helmann, 2011). The activation of this stimulon is complemented and overlaps with regulons controlled by SigB (general stress response), PerR (peroxide stress), OhrR (organic peroxide stress), AdhR (formaldehyde stress), and YodB (disulfide stress) to produce factors that detoxify metabolic intermediates and reactive oxygen species, prevent protein damage, and protect DNA from accumulating lesions (Antelmann and Helmann, 2011).

Using RNA extracted from stationary-phase *B. subtilis* cultures exposed to either 0 or 1 mM of the protein oxidant diamide, we found that nearly half of the genes in the transcriptome and several biological functions were expressed differentially in the absence of Mfd. Pathway enrichment analysis indicated that the absence of Mfd dysregulates expression of genes affecting biological processes that include translation, endospore formation, and flagellar motility. This dysregulation associated with phenotypic changes in growth in a defined medium, motility, and sensitivity to antibiotics. In addition to identifying pathways affected by Mfd expression, we were also interested in investigating potential gene targets that work in concert with Mfd to confer protection against disulfide stress. Genetic interaction experiments showed that the effect of Mfd on the cellular response to diamide was complex. For example, disruption of *sodA*, which encodes superoxide dismutase, abrogated the ability of cells to survive exposure to diamide, but overexpression of *mfd* in the SodA^–^ background restored it. Our genetic interaction assays uncovered, additive, epistatic, and suppressor effects on the response to disulfide stress.

In conclusion, the results presented here expand the roles of Mfd in the cell beyond transcription-coupled repair and suggest that this factor is a global modulator of transcription with profound effects on bacterial physiology and adaptation to stress.

## Materials and Methods

### Bacterial Strains and Growth Conditions

The parental strain, YB955, is a prophage- “cured” *B. subtilis* strain 168 derivative that contains the point mutations *metB5*, *hisC952*, and *leuC427*. *B. subtilis* strains employed in this study (Table 1) were routinely isolated on tryptic blood agar base (TBAB) (Acumedia Manufacturers, Inc., Lansing, MI, United States), and liquid cultures were grown in Penassay broth (PAB) (antibiotic medium 3, Difco Laboratories, Sparks, MD, United States) supplemented with 1X Ho-Le trace elements (*Methods for General and Molecular Bacteriology.* Washington, D.C.: American Society for Microbiology) (1994). When required, tetracycline (Tet; 10 μg⋅mL^–1^), spectinomycin (Sp; 100 μg⋅mL^–1^), ampicillin (Amp; 100 μg⋅mL^–1^), chloramphenicol (Cm; 5 μg⋅mL^–1^), erythromycin (Em; 1 μg⋅mL^–1^) or isopropyl-β-D-thiogalactopyranoside (IPTG; 1 mM) were added to media.

**TABLE 1:** Strains and plasmids used in this study.

Strain name	Genotype	Reference or source
YB955	*hisC952 metB5 leuC427* xin-1 Spβ^SENS^	Sung and Yasbin, 2002
YB9801	*hisC952 metB5 leuC427* xin-1 Spβ^SENS^ *mfd:tet*	Ross et al., 2006
PERM1134	*hisC952 metB5 leuC427* xin-1 Spβ^SENS^ *mfd:tet amyE:pHS-mfd*	Ramirez-Guadiana et al., 2013
HAM800	*hisC952 metB5 leuC427* xin-1 SpβSENS *ohrR:erm*	This study
HAM801	*hisC952 metB5 leuC427* xin-1 Spβ^SENS^ *sigB:erm*	This study
HAM802	*hisC952 metB5 leuC427* xin-1 Spβ^SENS^ *perR:erm*	This study
HAM803	*hisC952 metB5 leuC427* xin-1 Spβ^SENS^ *yodB:erm*	This study
HAM806	*hisC952 metB5 leuC427* xin-1 Spβ^SENS^ *cysK:erm*	This study
HAM807	*hisC952 metB5 leuC427* xin-1 Spβ^SENS^ *ssuC:erm*	This study
HAM810	*hisC952 metB5 leuC427* xin-1 Spβ^SENS^ *cysK:erm mfd:tet*	This study
HAM812	*hisC952 metB5 leuC427* xin-1 Spβ^SENS^ *ssuC:erm mfd:tet*	This study
HAM826	*hisC952 metB5 leuC427* xin-1 Spβ^SENS^ *bshA:erm*	This study
HAM827	*hisC952 metB5 leuC427* xin-1 Spβ^SENS^ *bshA:erm mfd:tet*	This study
HAM828	*hisC952 metB5 leuC427* xin-1 Spβ^SENS^ *bshB1:erm*	This study
HAM829	*hisC952 metB5 leuC427* xin-1 Spβ^SENS^ *bshB1:erm mfd:tet*	This study
HAM830	*hisC952 metB5 leuC427* xin-1 Spβ^SENS^ *sodA:erm*	This study
HAM831	*hisC952 metB5 leuC427* xin-1 Spβ^SENS^ *sodA:erm mfd:tet*	This study
HAM832	*hisC952 metB5 leuC427* xin-1 Spβ^SENS^ *yodB:erm mfd:tet*	This study
HAM833	*hisC952 metB5 leuC427* xin-1 Spβ^SENS^ *ykuV:erm mfd:tet amyE:pHS-mfd*	This study
HAM834	*hisC952 metB5 leuC427* xin-1 Spβ^SENS^ *ohrR:erm mfd:tet amyE:pHS-mfd*	This study
HAM835	*hisC952 metB5 leuC427* xin-1 Spβ^SENS^ *perR:erm mfd:tet amyE:pHS-mfd*	This study
HAM836	*hisC952 metB5 leuC427* xin-1 Spβ^SENS^ *sodA:erm mfd:tet amyE:pHS-mfd*	This study
HAM837	*hisC952 metB5 leuC427* xin-1 Spβ^SENS^ *yodB:erm mfd:tet amyE:pHS-mfd*	This study
JG001	*hisC952 metB5 leuC427* xin-1 Spβ^SENS^ *polYB:erm*	This study
JG002	*hisC952 metB5 leuC427* xin-1 Spβ^SENS^ *ohrB:erm*	This study
JG003	*hisC952 metB5 leuC427* xin-1 Spβ^SENS^ *bstA:erm*	This study
JG008	*hisC952 metB5 leuC427* xin-1 Spβ^SENS^ *ohrR:erm mfd:tet*	This study
JG009	*hisC952 metB5 leuC427* xin-1 Spβ^SENS^ *perR:erm mfd:tet*	This study
JG010	*hisC952 metB5 leuC427* xin-1 Spβ^SENS^ *sigB:erm mfd:tet*	This study
JG011	*hisC952 metB5 leuC427* xin-1 Spβ^SENS^ *bstA:erm mfd:tet*	This study
JG013	*hisC952 metB5 leuC427* xin-1 Spβ^SENS^ *ohrB:erm mfd:tet*	This study
JG014	*hisC952 metB5 leuC427* xin-1 Spβ^SENS^ *polYB:erm mfd:tet*	This study
KL101	*hisC952 metB5 leuC427* xin-1 Spβ^SENS^ *cypC:erm*	This study
KL105	*hisC952 metB5 leuC427* xin-1 Spβ^SENS^ *aldY:erm*	This study
KL201	*hisC952 metB5 leuC427* xin-1 Spβ^SENS^ *aldY:erm mfd:tet*	This study
KL205	*hisC952 metB5 leuC427* xin-1 Spβ^SENS^ *cypC:erm mfd:tet*	This study
BH001	*hisC952 metB5 leuC427* xin-1 Spβ^SENS^ *ykuV:erm*	This study
BH002	*hisC952 metB5 leuC427* xin-1 Spβ^SENS^ *ykuV:erm mfd:tet*	This study

### Construction of Mutant Strains

To construct single mutant strains, genomic DNA was isolated from the corresponding BKE (Bacillus Knockout Erythromycin collection; Koo et al., 2017) strains using the Wizard^®^ Genomic DNA Purification Kit (Promega, Madison, WI). Of note, the BKE gene deletion constructs are designed to minimize functional interference on the flanking open reading frames. Isolated genomic DNA was then transformed into YB955 using the competence procedures for *Bacillus* described previously (Yasbin et al., 1975). Briefly, YB955 was grown to T_90_, 90 min after the cessation of growth (stationary phase), in GM1 broth (0.5% dextrose, 0.1% yeast extract, 0.2% casein hydrolysate, essential amino acids 50 μg/mL, 1X Spizizen salt solution and then diluted 10-fold into GM2 broth (GM1 broth plus 50 μM CaCl_2_, 250 μM MgCl_2_). After 1 h of incubation at 37°C with aeration, genomic DNA (100 ng) was added. Cells were plated on TBAB containing 5 μg/mL erythromycin to select for the BKE allele. Transformants were confirmed by PCR.

To construct double mutant strains, genomic DNA from YB9801 (Mfd^–^) was isolated and transformed into *B. subtilis* strains with single mutations as described above (Table 1). Cells were plated on TBAB containing 10 μg/mL tetracycline to select for the *mfd*^–^ allele and 5 μg/mL erythromycin for maintenance of the BKE allele. Transformants were confirmed by PCR with specific oligonucleotide primers.

To construct the *mfd*-restored strains, BKE or PERM1134 DNA was isolated and transformed as described above. Of note, in this construct, the *mfd* gene is expressed from an IPTG-dependent promoter, and previous experiments showed that IPTG amendment results in restoration of Mfd functions to levels above those observed in the parent strain (Martin et al., 2019). Cells were plated on TBAB containing 100 μg/mL spectinomycin, 10 μg/mL tetracycline, and 5 μg/mL of erythromycin. Transformants were confirmed by PCR.

### RNA Sequencing and Differential Gene Expression Analysis

Briefly, a single colony was used to start a 2-mL PAB overnight culture. The next morning 0.5 mL was used to start a 15 mL PAB culture. Cultures were grown in flasks containing PAB and Ho-Le trace elements with aeration (250 rpm) at 37°C until 90 min after the cessation of exponential growth [designated T_90_ (90 min after the time point in the culture when the slopes of the logarithmic and stationary phases of growth intercepted)]. Growth was monitored with a spectrophotometer measuring the optical density at 600 nm (OD_600_). At T90, cultures were divided, and half were treated with 1 mM diamide, and incubated for another 2 h.

Total RNA from three biological replicates was harvested from cells differing in Mfd proficiency and treated or untreated with diamide, using the MP FastRNA Pro Blue Kit, and treated with DNase to remove residual DNA (Waltham, MA). Ribosomal RNA was removed by Ribo-Zero Magnetic Kit for Gram-Positive Bacteria, and the remaining RNA was then fragmented. The RNA samples were reverse transcribed into cDNA and sequenced. High quality sequence reads were generated using a HiSeq platform (2 × 150 bp read length) and aligned using HISAT2 (v 2.1.0) short read aligner to the latest version of reference in the Pubmed database (GCA_000009045.1). All sequence data were deposited to the NCBI SRA database under the bio project ID PRJNA673980. Read counts were generated using featureCounts (v1.6.2). Gene expression was quantified as the total number of reads uniquely aligning to the reference, binned by annotated gene coordinate. Differential gene expression and related quality control analyses was determined using the Bioconductor package DESEQ2. Normalization of raw read counts was performed by a scaling method implemented within DESEQ2 package, which accounts for differences in sequencing depth and composition. Differential expression of pairwise comparisons (of the different conditions) was assessed using the negative binomial test with a Benjamini–Hochberg false discovery rate (FDR) adjustment applied for multiple testing corrections.

### Growth Assays in Complex and Defined Media

A single colony was used to start a 2-mL PAB (complex) overnight culture. To start cultures for the growth curve, the OD_600_ for each overnight culture was measured. Cells were diluted to an OD_600_ of 0.4 for each strain and replicate. Then in a 96-well flat-bottom plate, 200 μL of PAB, 10 μL of the diluted overnight cultures, and either 0 mM or 0.5 mM diamide were mixed. Complemented strains were supplemented with 1 mM IPTG to induce expression of *mfd*. The growth curve was incubated at 37°C with shaking on the Synergy HTX plate reader. Readings were taken every 5 min for 16 h. Each strain was replicated at least nine times. For the assay in defined medium, we used Spizizen medium with histidine, methionine, and leucine supplements (the test strains carry these three auxotrophic markers) (Spizizen, 1958; Sung and Yasbin, 2002).

### Motility Assay

To test for flagellum-based motility, we conducted experiments in media containing 0.7 and 0.3% agar concentrations. We examined the spread of colonies of Mfd derivative strains on 0.7% TBAB containing IPTG and measured the colony diameter at 0, 3, and 6 days after inoculation. A single colony was used to start a 2-mL PAB overnight culture. The next morning, the OD_600_ for each overnight culture was measured. Cells were diluted to an OD_600_ of 0.1 for each strain and replicate. 10 μL of the cell dilution was spotted on the TBAB plates containing (0.7% agar) and incubated at 37°C lid-side up, as previously described (Patrick and Kearns, 2009). We spotted three strains per plate. At least three biological replicates were completed. Assays using 0.3% agar were conducted similarly. However, we measured swimming diameter 10 h after inoculation. Measurements were analyzed by ANOVA, the differences between means were tested using the LSD test at *P* ≤ 0.05 and *P* ≤ 0.01. See statistical analysis for more details.

### Minimal Inhibitory Concentration Assays

BioMerieux antibiotic strips containing a gradient of concentrations were used to test the effects of Mfd on sensitivity to linezolid, ampicillin, rifampicin, trimethoprim, and daptomycin. Protocols for preparation of cultures and media were followed according to manufacturer’s instructions.

### Statistical Analysis

ANOVA was used to test for differences between means. When ANOVA indicated statistical significance between treatments, we used the Least Significant Difference (LSD) method at *P* ≤ 0.05 or *P* ≤ 0.01. ANOVA (complete randomized design) and LSD analyses were conducted using the IBM SPSS 27 software and the statistical package in the GraphPad Prism 9 graphing software. Mean comparisons were conducted in pairwise combinations, and statistically significant differences between any two means were denoted by assigning different letters. We assigned “a” to the means that were not significantly different from the mean with the highest value, “b” to means that were different from the “a” group, and so on. A similar analysis was used in the study that first documented the effect of Mfd on tolerance to diamide (Martin et al., 2019).

### Pathway Analysis by Gene Enrichment

Gene enrichment or pathway analyses was performed using the ClueGO plug-in module of the Cytoscape software program, which annotates a list of genes to biological functions (gene ontologies) in a hierarchical way against an annotated genome (Bindea et al., 2009). Lists of genes down or up regulated in the absence of Mfd and in conditions of diamide exposure were used as input into Cytoscape and analyzed for overrepresented gene ontologies. Also, Kappa statistics, within the ClueGO plug-in, were calculated to link gene networks. For example, the list of genes that were downregulated mapped to 13 gene ontology terms and were grouped into 3 groups based on Kappa scores (translation, cell differentiation, and protein folding (Supplementary Material). These grouping results were obtained using the ClueGO plug-in of Cytoscape with the following settings: biological functions for gene ontologies, medium network specificity, GO tree interval with level 3 as minimum and level 8 as maximum, enrichment (right -sided hypergeometric test) with a pV value of 0.05 or less with the Bonferroni correction, and a GO term/pathway network connectivity (Kappa Score threshold) of 0.4.

## Results

### Mfd Modulates Global Transcription in Stationary-Phase and During Disulfide Stress in *B. subtilis* Cells

**FIGURE 1:**
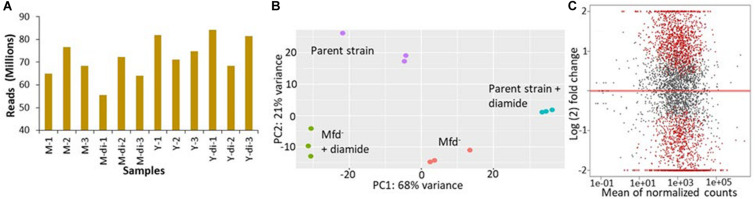
Mfd alters the transcriptome in stationary-phase cells in the presence and absence of diamide. **(A)** Mapped sequencing reads generated by independent RNA samples from M: *mfd* mutant, Y9801; Y: YB955, the parental strain; and di: exposure to diamide. **(B)** Principal component analysis of gene expression results from independent samples (*n* = 3/strain and treatment combination). **(C)** Gene expression pairwise comparison between the parent, YB955 (reference) and the *mfd* mutant, YB9801, in untreated cells. Each dot represents fold change value in expression of a single gene. Red dots indicate significant change in expression.

We conducted transcriptomic analysis assays in stationary-phase cells untreated or treated with 1 mM diamide of the parental (YB955) and Mfd^–^ (YB9801) strains. We used 3 independent cultures for each condition, which totaled 12 independent observations. The overall results showed that sufficient depth of coverage was established with reads mapping to genes uniquely (mapping to a single location), that expression patterns between independent cultures clustered according to experimental conditions. The results also showed that almost half of the genome was differentially regulated by Mfd and as affected by diamide exposure. This result was striking (Figures 1A–C). Interestingly, the pairwise comparison between the parent and the Mfd mutant in the absence of diamide exposure showed that mRNA levels of a significant number of genes were dysregulated in the absence of Mfd (Figure 1C). These results suggested that Mfd has a major impact on the transcription profile of the cell in stationary-phase conditions. Pairwise comparisons for changes in gene expression between the parent strain and the Mfd^–^ mutant in untreated and diamide-treated cells are presented in Supplementary Table S1.

In the untreated condition, 1,066 genes were downregulated in the absence of Mfd. The values in fold change expression [Log_(__2__)_ in these genes were from −0.39 to −3.55]. Pathway-enrichment analysis showed that genes in thirteen biological functions were downregulated in the absence of Mfd. Three major biological pathways were highlighted by Kappa scores: protein folding (Gene Ontology ID 006457), cell differentiation (GO ID 0030154- including spore synthesis and germination), and translation (GO ID 0006412) (Supplementary Table S2 and Supplementary Figure S4). On the other hand, expression of 931 genes was upregulated, and fold expression (Log_2_) values ranged from 0.42 to 7.78. Thirty-six biological functions were disproportionately upregulated in the absence of Mfd. These 36 biological functions were coalesced into 10 major groups by Kappa scores and include pentose metabolism (GO ID 0019321), cellular nitrogen and organonitrogen compound biosynthetic processes (GO ID 19001566, GO ID 004271), phosphorus metabolism (GO ID 0006793), toxin metabolism (GO ID 0009404), and transport of carbohydrates and organics (GO ID 0008643 and 0071702), ribonucleoside monophosphate and pyrimidine-containing compound biosynthesis (GO ID 0009156 and GO ID 0072528), and flagellum dependent motility (GO ID 0001539) (Supplementary Table S3 and Supplementary Figure S5).

In the diamide treated condition, Mfd deficiency resulted in the down-regulation of 1,365 genes. Enrichment pathway analysis showed that genes in 23 gene ontologies were down regulated in the absence of Mfd. These biological functions were grouped by Kappa scores into the ones observed in the untreated condition, proteolysis (GO ID 0006508) and glutamine biosynthesis (GO ID 0009084), isoprenoid metabolism (GO ID 0006720), antibiotic metabolism (GO ID 0016999), carboxylic acid metabolism (GO ID 0019752), and protein metabolism (GO ID 0019538). The fold change in gene expression (Log_2_) ranged from −0.4 to −6.1 (Supplementary Table S4 and Supplementary Figure S6). In addition, the Mfd^–^ background displayed up-regulation of 1,040 genes, the enrichment analysis showed 24 gene ontologies that included inosine monophosphate (IMP) biosynthesis (GO ID 0006188), cell projection organization (GO ID 0030030), flagellum motility and chemotaxis (GO ID 0001539, GO ID0006935), transmembrane and sodium transport (GO ID 0006814 and 0055085), and cellular nitrogen biosynthesis (GO ID 0044271) (Supplementary Table S5 and Supplementary Figure S7). In summary, these results showed that Mfd affects many biological processes in stationary-phase *B. subtilis*.

### Mfd Influences Sensitivity to Antibiotics, Growth in Defined Medium, and Motility

Transcriptomic results indicated that loss of Mfd caused dysregulation of global gene expression and prompted us to test for growth and other phenotypes associated with the biological functions that were highlighted by the pathway enrichment analysis (ribonucleoside phosphate biosynthesis, and motility). Also, 108 essential genes were dysregulated in Mfd^–^ cells (Supplementary Table S1). The genes *liaF*, *liaR*, and *liaS*, which are activated during oxidative and cell-envelop stress (Radeck et al., 2017), were differentially expressed by the loss of Mfd. Mfd affected expression of *rpoB* and *rpoE*. These genes encode the β and Δ subunits of the RNA polymerase (Boor et al., 1995). Given these observations, and previous reports of Mfd effects on gene expression of amino acid biosynthesis, we were motivated to investigate the ability to grow on a defined medium (Spizizen medium) and measure minimal inhibitory concentrations (MIC) for ampicillin, linezolid (controls), trimethoprim (thymidine synthesis), daptomycin (cell membrane), and rifampicin (transcription) and in cultures of the parent, Mfd^–^, and Mfd^–^ carrying an *mfd*-overexpressing construct in the *amyE* chromosomal locus.

**FIGURE 2:**
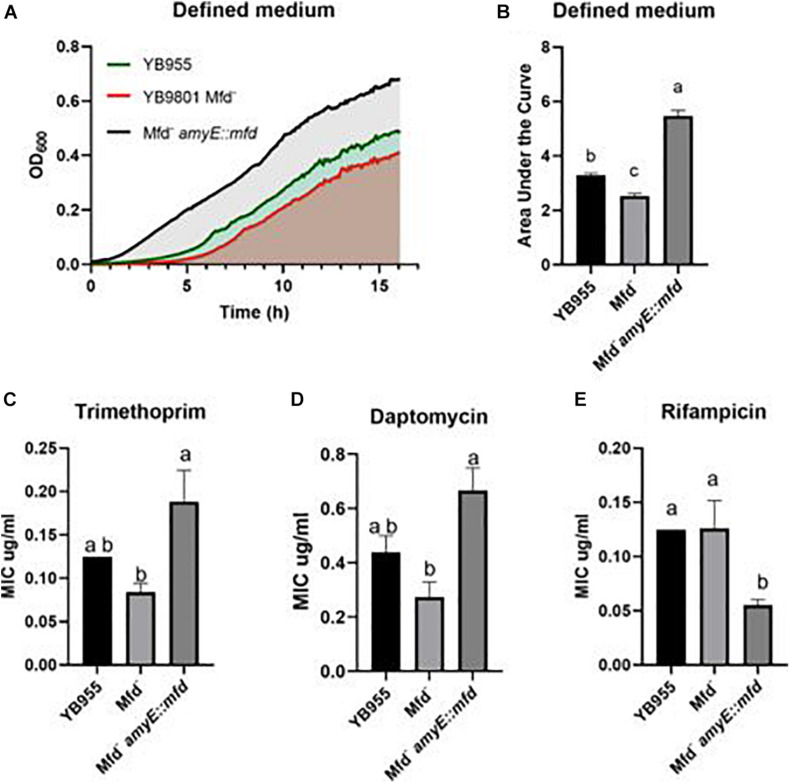
Effects of Mfd on growth in defined medium and minimal inhibitory concentrations MIC to antibiotics. **(A)** Average OD_600_ readings of strains differing in Mfd measured by a plate reader (*n* = 9). **(B)** Average area under the curve as calculated by the R package, Growthcurver, of growth curves from **(A)**. **(C–E)** Average MIC of strains differing in Mfd determined by ETEST strips (*n* = 3). AUC was analyzed by ANOVA, and means were separated using the Least Significant Difference (LSD) test. Lower case letters distinguish significant differences between means. “a,” “b,” “c” and onward are significantly different mean groups at *P* ≤ 0.05. Bars on top the columns represent SEM.

We measured growth in defined medium for 16 h and showed similar values for doubling time, cell density, and growth lag between the parent and Mfd^–^ cells (Figure 2A). However, the *mfd*-overexpressing strain showed marked differences in growth lag and cell density compared to the parent or Mfd^–^ cells (Figure 2A). To better quantify overall growth dynamics, which integrate doubling time and cell density, we used the area under the curve, as calculated by the R-based program GrowthCurver (Sprouffske and Wagner, 2016), to measure the effect of Mfd on growth. The area under the curve (AUC) mean values indicated that the *mfd*-overexpressing cells displayed a significant increase in growth compared to the parent, and the parent’s AUC was significantly higher than the Mfd^–^ strain (Figure 2B).

All the strains were equally sensitive to ampicillin (lowest concentration—0.016 μg/ml) and resistant to linezolid (highest concentration—256 μg/ml). Interestingly, the loss of Mfd resulted in lower, but not significant, MIC values than the parent for trimethoprim and daptomycin (Figures 2C,D). However, overexpression of *mfd* produced significant increases in MIC values when compared to the Mfd^–^ strain (∼2- and ∼3-fold for trimethoprim and daptomycin, respectively). On the other hand, Mfd^–^ cells did not show significant differences to rifampicin when compared to the parent, but the cultures that overexpressed *mfd* were more sensitive than the parent (Figure 2E).

**FIGURE 3:**
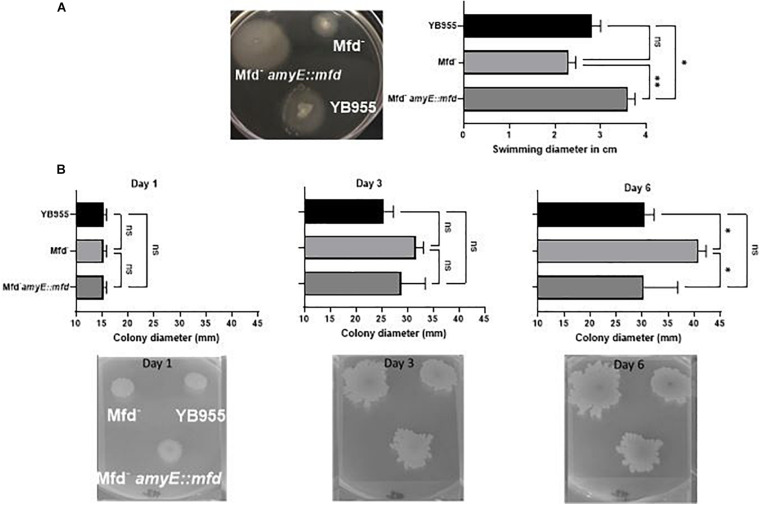
Deficiencies in Mfd reduce swimming motility and increase colony spread. **(A)** Effect of Mfd on swimming motility. Mfd derivatives were spot inoculated on 0.3% TBAB agar (*n* = 3). Swimming diameter was measured 10 h after inoculation analyzed by ANOVA; means were separated using the LSD test. **(B)** Effect of Mfd on colony spread on 0.7% TBAB agar. ns, *, ** denote no significant differences, different at *P* ≤ 0.05, and *P* ≤ 0.01, respectively.

Cells lacking Mfd showed upregulation of 15 genes (*flgC, flgK, flgL, flhO, flhP, fliE, fliI, fliJ, fliK, fliL, fliM, fliY, hag, motA, and motB—*Supplementary Table S1) controlling flagellum-based motility, which prompted us to test if there was a difference in motility-associated phenotypes of *B. subtilis* differing in Mfd. Interestingly, Mfd^–^ cells upregulated the expression of *srfAB*, *srfAC*, *srfAD* (Supplementary Table S1), which contain genes for surfactin biosynthesis and factors required for genetic competence (Dubnau, 1991; Nakano et al., 1991; Cosmina et al., 1993; Fuma et al., 1993). We tested Mfd derivatives on TBAB plates containing 0.7% and 0.3%, and incubated at 37°C. Colonies lacking Mfd displayed increased spread after 3 days, and the Mfd-defective cells were significantly increased in colony spread 6 days after inoculation on 0.7% agar compared to the parent cells and those that overexpressed *mfd* (Figure 3). The swimming motility assay showed significant differences between strains. The *mfd* mutant showed a diminished ability to swim compared to the parent strain, albeit not significantly. However, *mfd* overexpression resulted in a significant increase in swimming motility compared to the parent and the *mfd* mutant. Altogether, these results suggest that Mfd affects fundamental aspects of bacterial physiology.

### Mfd Modulates the Response to Disulfide Stress via Complex Genetic Interactions in *B. subtilis*

**FIGURE 4:**
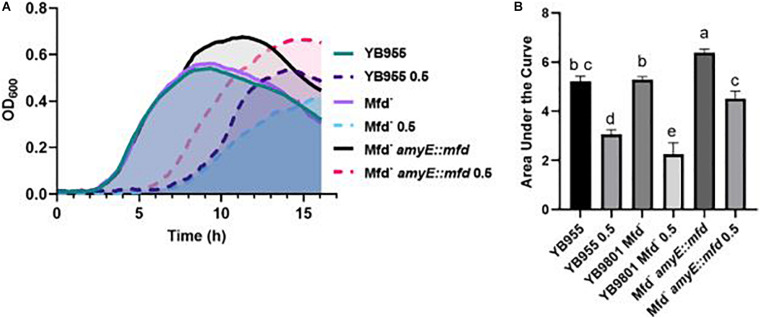
Effects of Mfd on growth in a complex medium and in the presence of diamide. **(A)** Average OD_600_ readings of strains differing in Mfd measured by a plate reader (*n* = 9). **(B)** Average area under the curve (AUC) as calculated by the R package, Growthcurver, of growth curves from **(A)**. AUC was analyzed by ANOVA, and means were separated using the Least Significant Difference (LSD) test. Lower case letters distinguish significant differences between means. “a,” “b,” “c” and onward are significantly different mean groups at *P* ≤ 0.05. Bars on top the columns represent SEM.

In addition to the experiments measuring growth in defined medium, sensitivity to antibiotics, and motility, we examined the effect of Mfd in stressed cells and in the context of protein oxidation. Our previous report showed that Mfd protected cells against disulfide stress, but not much is known about how this factor operates to provide cells such protection (Martin et al., 2019). Growth in complex broth in the absence and presence of diamide was followed by OD_600_ measurements every 5 min for 16 h. The growth dynamics of the parent and Mfd^–^ strains were indistinguishable in the untreated cells, and both genetic backgrounds showed a similar growth lag in the presence of diamide (Figure 4A). However, the Mfd^–^ cells displayed an increase in doubling time during mid exponential growth and a decrease in cell density compared to the parent strain in the presence of diamide. Cells that overexpressed *mfd* displayed the same growth rate but an increase in cell density compared to the parent strain in untreated conditions (Figure 4A). Strikingly, the *mfd*-overexpressing cells showed a shorter growth lag and a higher cell density than the parent strain, but both strains showed a similar doubling time in the treated cells (Figure 4A). The AUC values showed that growth in untreated cells was significantly different among the tested strains and that the *mfd*-overexpressing cells responded better than the parent and Mfd^–^ counterparts (*P* ≤ 0.05) (Figure 4B). Overexpressing *mfd* increased tolerance to diamide levels significantly better than in the parent and Mfd^–^ counterparts. In fact, the response in the *mfd*-overexpressing strain was like the one observed in the untreated parent strain. The Mfd^–^ cells displayed the lowest tolerance to diamide amongst the tested strains (Figure 4B). The results observed in untreated and diamide-treated cells suggest that Mfd controls the physiology of cells experiencing nutrient-limiting conditions and the response to disulfide stress.

To better understand the role of Mfd in the response to disulfide stress, we examined the genetic interactions between the Mfd factor and those genes that are either affected in expression by Mfd, are known transcription factors that control gene expression during oxidative damage, or are components of cysteine and sulfur metabolism, which produce compounds important for disulfide and electrophile stress tolerance (Hochgräfe et al., 2007; Gaballa et al., 2010). We selected 15 genes to generate single knockouts in our parental strain, YB955 (Table 1 and Supplementary Table S6). Then, we produced double knockouts of *mfd* and each of the 15 selected genes by transforming genomic DNA from our *mfd* mutant strain, YB9801, into the single knockout. Also, for 5 of the 15 genes under study, we generated strains in which the double knockout was transformed with genomic DNA from our *mfd*-restored strain, PERM1134. This process generated three types of strains: (i) single gene knockouts, (ii) double knockout combinations of *mfd* and each of the 15 genes, and (iii) double knockouts with an *mfd* overexpressing construct ectopically placed in the chromosome.

**FIGURE 5:**
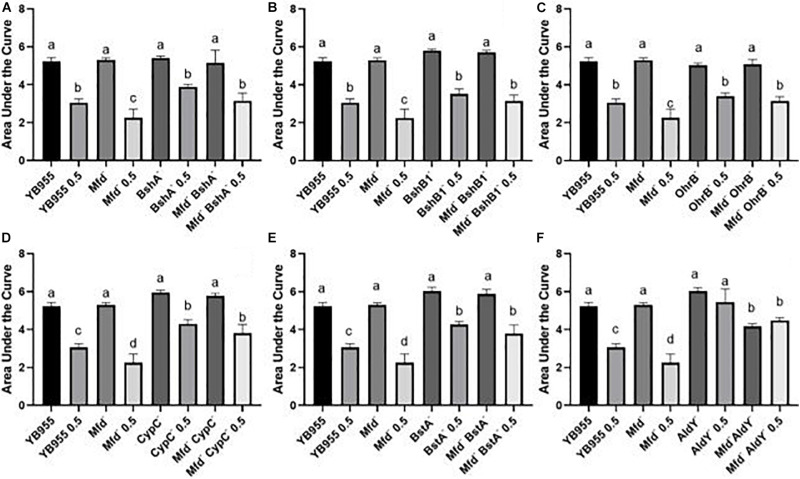
Growth dynamics of single- and double-gene-deletion strains in the presence and absence of diamide show gene suppressors and recessive interactions. Average area under the curve as calculated by the R package, Growthcurver, of growth curves from Supplementary Figure S1. Each graph represents an independent trial of single and combined mutations of **(A)** *bshA*, **(B)** *bshB1*, **(C)** *ohrB*, **(D)** *cypC*, **(E)** *bstA*, and **(F)** *aldY*, and *mfd*, and each AUC mean is based on *n* = 9. AUC was analyzed by ANOVA, and means were separated using the Least Significant Difference (LSD) test. Lower case letters distinguish significant differences between means. “a,” “b,” “c” and onward are significantly different mean groups. Bars on top the columns represent SEM.

We conducted growth assays in the presence and absence of 0.5 mM of diamide and used the area under the curve to measure the growth response and tolerance to diamide in all the tested strains. Strains with single mutations in *bshA, bshB1*, and *ohrB* showed a similar growth response and tolerance to diamide to the parent (Figures 5A–C and Supplementary Figure S1). The *bshA*, and *bshB1* gene products are components of the bacillithiol biosynthesis, and *ohrB* encodes a peroxiredoxin (Völker et al., 1998; Fuangthong et al., 2001; Gaballa et al., 2010). These results suggest that the single contributions of these factors to disulfide stress tolerance are negligible (Figures 5A–C). However, the mean response in tolerance to diamide in cells with mutations in *mfd* and each of these three factors were statistically the same as the one observed in the parent. These results suggest that mutations in *bshB1*, *bshA*, and *ohrB* suppress the effect of *mfd* on disulfide stress.

Mutations in *cypC*, *bstA*, and *aldY* did not affect growth, but exerted increased tolerance to diamide when compared to the parent or Mfd mutant (Figures 5D–F and Supplementary Figure S1). The factors coded by these genes are involved in synthesis of lipopeptides and bacillithiol, and tolerance to alcohol exposure (Matsunaga et al., 1999; Petersohn et al., 1999; Perera et al., 2018). Interestingly, the levels of increased tolerance to diamide in the CypC^–^ and BstA^–^ backgrounds were unaffected by mutations in *mfd*. These results suggest that the loss of these factors alters the cell physiology to better withstand disulfide stress and exert an epistatic effect on *mfd*. Mutations in *aldY* did not affect growth but increased tolerance to diamide compared to the parent. In fact, there was no difference between treated and untreated AldY^–^ cells. The combined mutations in *mfd* and *aldY* resulted in diminished growth compared to the parent or *aldY* mutant, suggesting that both functions are required for optimal growth. Interestingly, the absence of both gene products showed similar AUC values in treated (Mfd^–^ AldY^–^ 0.5) and untreated cells (Mfd^–^ AldY^–^), indicating that deficiencies in AldY desensitize cells to diamide (Figure 5F).

**FIGURE 6:**
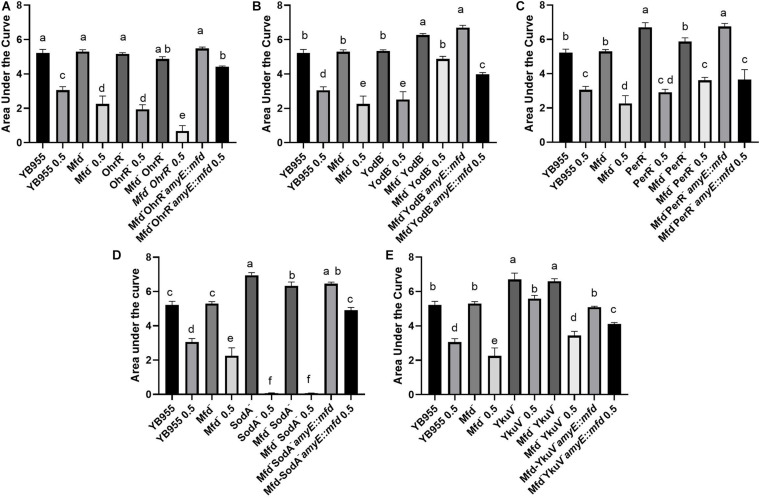
Overexpression of *mfd* can rescue or sensitize cells to diamide. Growth dynamics of single- and double-gene-deletion and overexpressing-*mfd* strains in the presence and absence of diamide. Average area under the curve as calculated by the R package, Growthcurver, of growth curves from Supplementary Figure S2. Each graph represents an independent trial of single and combined mutations of **(A)** *ohrR*, **(B)** y*odB*, **(C)** *perR*, **(D)** *sodA*, and **(E)** *ykuV*, and *mfd*. Each AUC mean is based on *n* = 9. AUC was analyzed by ANOVA, and means were separated using the Least Significant Difference (LSD) test. Lower case letters distinguish significant differences between means. “a,” “b,” “c” and onward are significantly different mean groups. Bars on top the columns represent SEM.

We conducted experiments that included overexpression of the Mfd factor in strains with mutations in transcription factors that affect the response to either oxidative or disulfide stress, as well as in strains with mutations in genes coding for a thiol-oxidoreductase and a superoxide dismutase (Inaoka et al., 1998; Fuangthong et al., 2001; Zhang et al., 2006). Mutating *ohrR* did not affect growth, but decreased tolerance to diamide treatment to the levels observed in the Mfd^–^ background (Figure 6A and Supplementary Figure S2). The results shown by the double inactivation of *ohrR* and *mfd* suggest that these genes contribute to diamide additively and, therefore, are part of different cellular pathways (Figure 6A). Restoring *mfd* with an overexpressing construct in the *ohrR mfd* mutant resulted in increased tolerance to diamide levels higher than the ones observed in the parent (Figure 6A). Deficiencies in YodB, a repressor that controls expression of genes that respond to disulfide stress (Chi et al., 2010), did not affect growth, but displayed decreased tolerance to diamide levels comparable to the Mfd^–^ strain. However, the combined effects of deficiencies in Mfd and YodB resulted in increased growth and tolerance to diamide, and overexpressing *mfd* in this background sensitized cells to it (Figure 6B and Supplementary Figure S2). Inactivation of *perR*, the gene that encodes the repressor that controls the response to peroxide (Bsat et al., 1998), increased growth but showed no differences in tolerance to protein oxidation compared to the parent strain. The response in the *mfd perR* strain suggests that a component of the increase in growth seen in the Per^–^ cells is dependent on Mfd; restoring this factor restored growth to the levels seen in the Per^–^ cells but did not change the response to diamide compared to the double mutant (Figure 6C and Supplementary Figure S2).

Genetically inactivating SodA increased growth but abrogated tolerance to protein oxidation, and a similar response was observed when this mutation combined with Mfd deficiencies in treated and untreated cells (Figure 6D and Supplementary Figure S2). So, the phenotype caused by the loss of function in SodA persisted in Mfd^+^ cells. This result suggests that the *sodA* gene has a recessive epistatic effect onto *mfd*. However, tolerance to diamide was restored to levels higher than the parent in the *sodA mfd* strain that overexpressed *mfd*. This indicates that excess Mfd in the cell can remodel physiology to bypass the absence of SodA. Interestingly, cells that lack YkuV, which encodes a thiol oxidoreductase, showed an increase in growth and tolerance to diamide compared to the parent, and the values in diamide tolerance seen in the double mutant (*mfd ykuV*) suggest a suppressor effect onto the *mfd* mutation. Overexpression of *mfd* restored growth to the parent levels and increased tolerance to diamide significantly compared to the double mutant (Figure 6E and Supplementary Figure S2). This result suggests that excess Mfd can function to increase tolerance to diamide independently of the suppressor effect exerted by the loss of YkuV (compare the means of the double mutant and the YkuV^–^ mutant overexpressing *mfd* in the presence of diamide—Figure 6E).

The experiments examining the pairwise interactions between *mfd* and *sigB* (general stress response; Petersohn et al., 1999), *ssuC* (aliphatic sulfonate transporter; Van Der Ploeg et al., 1998), *polYB* (translesion synthesis polymerase; Duigou et al., 2004), and *cysK* (cysteine synthase; Van Der Ploeg et al., 2001); upregulated in cells exposed to diamide (Leichert et al., 2003) showed that single mutations in the latter four genes resulted in an increase in growth and tolerance to protein oxidation (Supplementary Figure S3). The double mutants *mfd sigB*, *mfd polY*, and *mfd ssuC* showed a similar response to the ones observed in the Mfd^+^ background, which suggests that strains with deficiencies in either SigB, PolYB, or SsuC respond to disulfide stress independently of Mfd. In contrast, the double mutant *mfd cysK* showed a decreased growth and tolerance to diamide compared to the CysK^–^ cells but expressed similar values in treated and untreated conditions (Supplementary Figure S3). This result suggests that the combined deficiencies in CysK and Mfd decrease growth but do not influence overall tolerance to diamide. Altogether, these results indicate that the effects of Mfd on the growth and the response to protein oxidation is complex and dependent on gene interactions, and that this factor can remodel cell physiology to sensitize or increase tolerance to disulfide stress.

## Discussion

Mfd affected expression of almost 2,000 genes in stationary-phase cells and several biological functions, even in the absence of disulfide stress. There are no reports on the effects of Mfd on overall cell physiology on stationary-phase bacterial cells. Mfd did not affect the growth dynamics in a complex medium compared to the parental strain (Figure 4). Nevertheless, Mfd^–^ cells showed differences in expression of 108 essential genes (83 were down regulated in the absence of Mfd—see Supplementary Table S1) that affect DNA replication, cell division, transcription, protein synthesis, and other biological functions. Given the changes in expression on essential genes, whose products are targeted by antibiotics, we tested Mfd derivatives for sensitivity to antibiotics that target cell envelope, transcription, and DNA replication. Also, previous reports on the effect of Mfd on gene transcription controlled by amino acid starvation (Gerhardt et al., 1994; Belitsky and Sonenshein, 2011) prompted us to test the different Mfd backgrounds for the ability to grow in a defined medium, in the absence of amino acid supplements. Mfd defects did not result in significant changes in MIC for trimethoprim, daptomycin, and rifampicin but its overexpression did (Figure 2). The results in growth in defined medium and antibiotic sensitivity support the idea that the Mfd-dependent changes in gene transcription alter cell physiology.

In the absence of Mfd, 65% of the genes for inosine monophosphate metabolism were upregulated. Other genes involved in nucleoside metabolism were affected by Mfd. These changes in gene expression are likely to induce a more sensitive state to trimethoprim; such sensitivity was reduced when *mfd* was overexpressed. Interestingly, activity of the ribonucleotide reductase, encoded by *nrdEF*, influences Mfd-dependent mutagenesis (Castro-Cerritos et al., 2017, 2018). The response in daptomycin correlated with low gene expression of the *liaRS* operon, which codes for factors that sense cell-envelope stress. It is worth noting that previous studies have implicated Mfd in the formation of mutations that confer resistance to antibiotics (Han et al., 2008; Ragheb et al., 2019); however, no reports associate Mfd-dependent changes in gene expression to antibiotic sensitivity. The effect of Mfd on rifampicin MIC is likely due to the changes in expression in *rpoB* and *rpoE* in the Mfd^–^ cells (Supplementary Table S1). Mfd is active during transcription elongation and at regions distant from the promoter (Haines et al., 2014) and processes backtracked RNAP into active transcription (Park et al., 2002). Perhaps, excess Mfd alters the equilibrium between the RNAPs engaged in initiation and elongation, and such alteration results in the increased sensitivity observed here.

Our assays showed that the absence of Mfd influenced motility (Figure 3). Our results on the colony spread on 0.7% were interesting. Our expectation was that Mfd derivatives would not differ in 0.7% agar because we worked with a domesticated strain; domesticated strains do not display swarming motility (Kearns et al., 2004; Patrick and Kearns, 2009), which is what we observed. However, we did note differences in colony spread after 6 days, suggesting that colony properties are affected by Mfd. The results from the RNASeq indicated that 15, but not all, structural genes for flagellum biosynthesis were upregulated in the absence of Mfd. Strikingly, Mfd^–^ cells were diminished in swimming. Flagellum biosynthesis is regulated in a complex manner in *B. subtilis* (Kearns et al., 2004; Mukherjee and Kearns, 2014), and the disparate upregulation of structural genes could have compromised the ability to swim in Mfd^–^ cells. To our knowledge, this is the first report that shows Mfd to be a factor that controls motility. Forty percent of genes involved in endospore formation were downregulated and suggest that the loss of Mfd affects sporulation. This assertion agrees with our previous report (Ramirez-Guadiana et al., 2013) and that of Koo et al. (2017) which showed that Mfd^–^ deficient cells display a 65% sporulation efficiency compared to Mfd^+^ cells. The decrease on sporulation efficiency could be the result of the combined deficiencies in gene expression and repair of oxidative damage during endospore formation in Mfd^–^ cells. Mfd^–^ cells with defects in DNA repair systems that target 8-oxo-G showed an exacerbated decrease in sporulation efficiency during oxidant exposure compared to the Mfd^+^ cells (personal communication Pedraza-Reyes).

The loss of Mfd significantly decreased growth in defined medium, and overexpressing of this factor produced an increase in the growth response in defined medium compared to the parent (Figure 2). On the other hand, growth in PAB (complex medium) showed no differences between the parent and the Mfd^–^ cells, but overexpression of Mfd conferred PAB cultures the ability to attain a higher cell density than the parent (Figure 4). These results suggest that Mfd facilitates gene expression of amino acid biosynthetic genes and a more efficient growth physiology in defined medium; in complex medium, Mfd-dependent changes in physiology allowed cells to continue doubling during the transition to nutrient scarcity. Strains containing gene deletions in *mfd* showed a slight growth defect in LB at 37°C, as measured by relative fitness (Koo et al., 2017). The results from our growth assays suggest that the biological consequences associated with deficiencies in Mfd express in cells nutritionally stressed or conditions in which amino acid biosynthesis is active.

The effects of Mfd on the overall response to diamide exposure were complex. In untreated conditions, several biological functions were affected by Mfd, and there were no consequences in the ability to grow on PAB, but it could be argued that the changes in gene expression observed in the untreated condition sensitize cells to protein oxidation. Contrastingly, the gene expression associated with the cellular functions that were affected in the absence of Mfd in treated cells only (protein degradation, glutamine, isoprenoid and carboxylic acid metabolism, antibiotic metabolism, inosine monophosphate biosynthesis, cell projection and organization, as well as transmembrane and sodium transport) are likely to compromise the response to disulfide stress. Future research will examine the specific contributions of those biological processes to sensitivity and adaptation to diamide exposure.

We studied the effects of Mfd on tolerance to protein oxidation further by measuring how genetic interactions influenced the cellular response to disulfide stress (Figures 5, 6 and Supplementary Figure S3). The results indicated different kinds of interactions, and the strains with single mutations in *bshA*, *bshB1*, *ohrB*, *cypC*, *bstA*, *sigB*, and *polYB* showed no significant differences compared to the strains with double mutations in *mfd bshA*, *mfd b*s*hB1*, *mfd ohrB*, *mfd cypC*, *mfd bstA*, *mfd sigB*, and *mfd polYB*. Then, the effects on growth in a complex medium and tolerance to diamide caused by the single mutations in these genes (*bshA*, *bshB1*, *ohrB*, *cypC*, *bstA*, *sigB*, and *polYB*) are Mfd-independent. Contrastingly, the effects of mutations in *aldY*, *ohrR*, *yodB*, *perR*, *sodA*, *ykuV*, *ssuC*, and *cysK* on growth in PAB (*aldY*, *yodB*, *perR*, *sodA*, and *cysK*) or tolerance to diamide (*aldY*, *ohrR*, *yodB*, *ykuV*, and *cysK*) were partially dependent on Mfd. The effect of Mfd overexpression on growth and tolerance to diamide was dependent on the genetic background tested (Figure 6). Interestingly, overexpression of *mfd* combined with mutations in *ohrR* and *sodA* resulted in increased tolerance to disulfide stress when compared to Mfd^–^ OhrR^–^ and Mfd^–^ SodA^–^ cells, respectively. We interpreted the *mfd*-overexpression results to mean that Mfd can function to modulate the response to diamide by different pathways.

Mfd is a multidomain enzyme that can translocate on DNA independently of its direct interactions with RNAP, and the Mfd number of molecules is estimated to range between 30 and 300 (Deaconescu et al., 2006; Ho et al., 2018; Le et al., 2018). Then, it is likely that a large fraction of the ∼2,000 genes affected by Mfd reported here are caused by indirect effects. However, the experiments that tested the effects of gene interactions and *mfd* overexpression on tolerance to diamide provide candidates for genes and cellular pathways whose expression are directly affected by Mfd. For example, tolerance to diamide in the *sodA* and *mfd sodA* mutants was abrogated but overexpression of *mfd* rescued it, which suggests an Mfd-dependent effect. Direct effects on genes can be identified by combining transcriptome experiments and assays that test Mfd interactions with the transcription machinery (NETSeq or ChIPSeq) (Ragheb et al., 2021). Similar assays were used recently to characterize transcriptional pauses as affected by NusG (Yakhnin et al., 2020). Mechanistically, *in vitro* studies have shown that Mfd can function as a transcription modulator (Selby and Sancar, 1995; Belitsky and Sonenshein, 2011; Le et al., 2018). Also, mutagenesis reports suggest that Mfd acts at highly transcribed regions that accumulate lesions and mediates the formation of mutations (Gomez-Marroquin et al., 2016). In the presence of Mfd, active transcription at catabolite-repressed genes is terminated at DNA sites distant from the promoter region and occupied by the *cre*-CcpA complex (DNA-protein repression block) (Zalieckas et al., 1998a). This mode of transcription termination operates at genes controlled by the CodY repressor (Belitsky and Sonenshein, 2011). On the other hand, if Mfd is required to process RNAP paused at intrinsic sites or pauses caused by non-B DNA structures back into active transcription, then loss of Mfd would result in a decrease of complete gene transcripts. *In vitro* studies demonstrated that non-B DNA structures can stall transcription (Tornaletti et al., 2008; Pandey et al., 2015).

A recent genome association report showed that expression of almost 380 genes were affected by Mfd during exponential growth; genes that were directly affected by this factor encode toxin-antitoxin modules (Ragheb et al., 2021). Further, those studies showed that overexpression of the *txpA*, *bsrH*, genes in Mfd^–^ cells decreased cell survival compared to the parent strain and led to propose a model in which Mfd directly interacts with RNAP to modulate transcription of regions with structured RNAs (Ragheb et al., 2021). Interestingly, our transcriptome assays were conducted in stationary-phase cells and showed no changes in expression of *txpA* and *bsrH* genes in untreated cells. However, Mfd^–^ cells treated with diamide were downregulated in transcription of those genes [–1.14 Log_(__2__)_ for *bsrH* and –1.79 for *txpA*], which suggests that Mfd-dependent modulation of transcription changes according to growth conditions.

## Conclusion

The results reported here and those in exponentially growing cells provide strong evidence for the concept that Mfd acts as global modulator of transcription and that the biological consequences associated with the loss of this factor manifest in stressed cells. From a mechanism standpoint, Mfd resolves pauses or blocks to transcription by dissociating a halted RNAP from the DNA or reactivating it into active transcription (Park et al., 2002; Deaconescu et al., 2006; Savery, 2007; Ho et al., 2018). Therefore, the loss of Mfd can increase or decrease the ability of an RNAP to complete a full transcript directly. Structural biology studies indicate that key features for Mfd to process a halted RNAP are its translocase activity and RNAP interactions (Smith and Savery, 2005; Brugger et al., 2020; Shi et al., 2020). While the conditions that lead Mfd to displace a halted RNAP (DNA-protein repression blocks and bulky-distortive DNA lesions) have been characterized, we know little about the type of events that is resolved by Mfd through realignment and reactivation of transcription complexes, particularly in stressed cells and at the systems’ level (Selby and Sancar, 1995; Saxowsky and Doetsch, 2006; Smith and Savery, 2008; Belitsky and Sonenshein, 2011).

In summary, this work demonstrates that Mfd has profound effects on the transcriptome and phenotypes of stationary-phase *B. subtilis.* The loss of Mfd results in deficiencies in transcription-coupled DNA repair, the cellular responses to nutrient deprivation, cell envelope stress, antibiotic exposure, and protein oxidation. Also, Mfd influences cell differentiation behaviors that include endospore formation (Ramirez-Guadiana et al., 2013; Koo et al., 2017) (personal communication/manuscript in review Pedraza-Reyes) and motility. Because Mfd is well-conserved in bacteria, it would not be surprising if the pleiotropic effects observed in *B. subtilis* extend to other bacterial species, including those that are pathogenic. Considering the mutagenesis functions of Mfd that confer fitness to stationary-phase cells and resistance to antibiotics (Pybus et al., 2010; Ragheb et al., 2019), our results suggest that this factor operates at the intersection between gene expression and mutagenesis that mediates adaptation to stress and bacterial evolution.

## Data Availability Statement

The datasets generated for this study can be found in the online repositories. The names of the repository/repositories and accession number(s) can be found below: https://www.ncbi.nlm.nih.gov/, ID PRJNA673980.

## Author Contributions

HM, AS, FS, MP-R, and ER planned the experiments. HM, TE, RH, JG, KL, DA-M, and ER performed the laboratory work. HM, AS, MP-R, and ER analyzed the data. HM and ER wrote the manuscript. AS, TE, RH, JG, KL, DA-M, FS, and MP-R edited the manuscript. All authors contributed to the article and approved the submitted version.

## Conflict of Interest

The authors declare that the research was conducted in the absence of any commercial or financial relationships that could be construed as a potential conflict of interest.
