# Supplementary figures and images for: Mfd Affects Global Transcription and the Physiology of Stressed Bacillus subtilis Cells
Source: Front Microbiol. 2021 Jan 28;12:625705. doi: 10.3389/fmicb.2021.625705 (PMC7885715; doi:10.3389/fmicb.2021.625705)

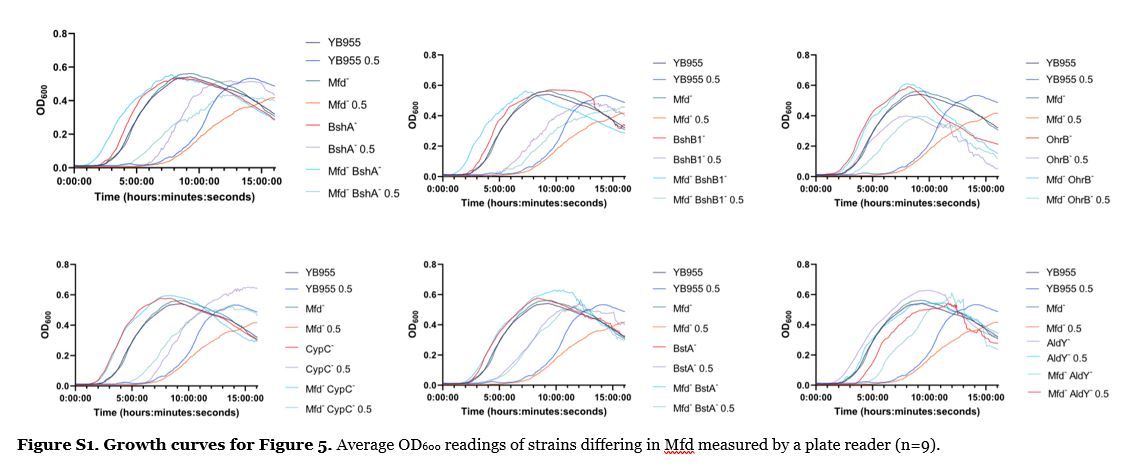

Supplement: Supplementary file 1 [file Image_1.JPEG]

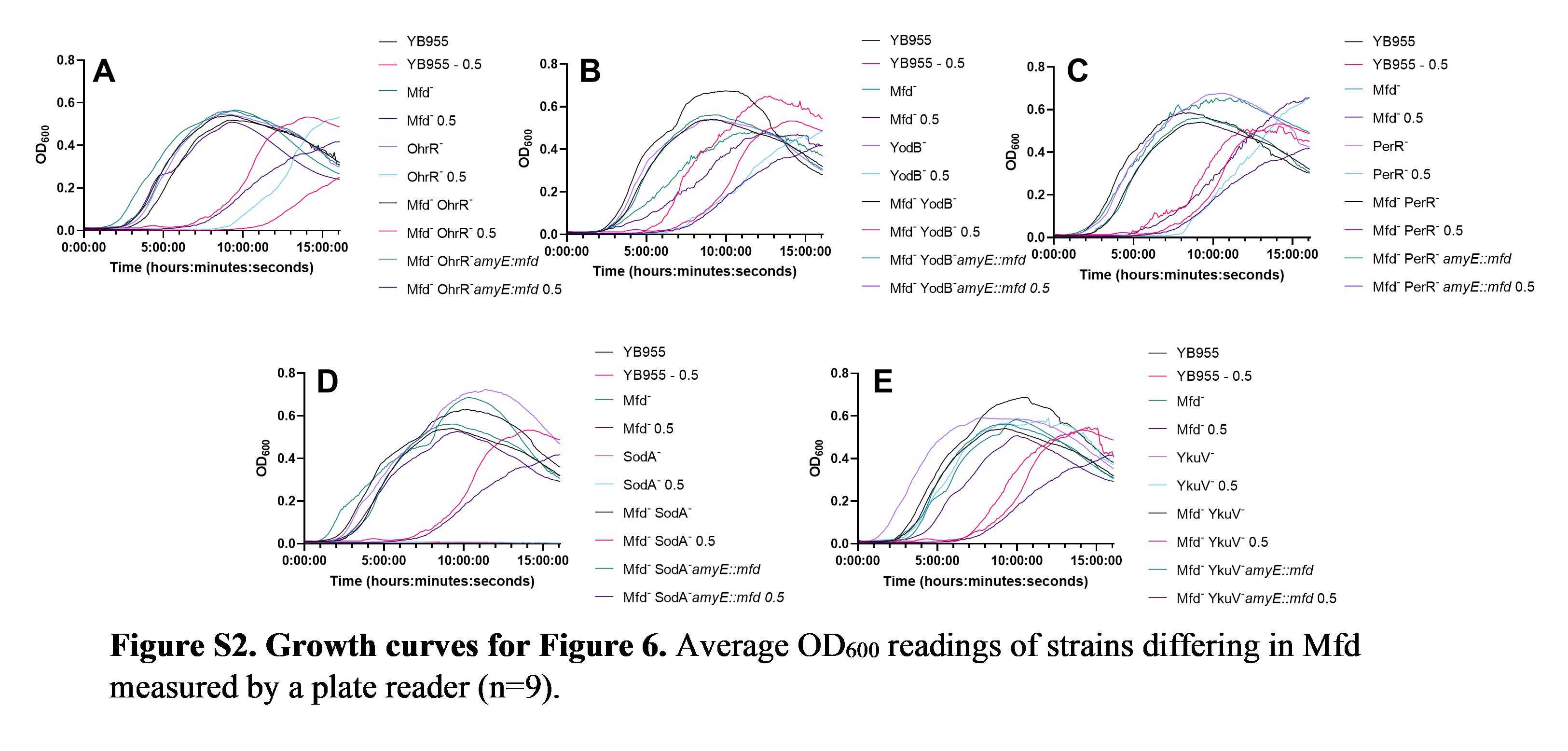

Supplement: Supplementary file 2 [file Image_2.JPEG]

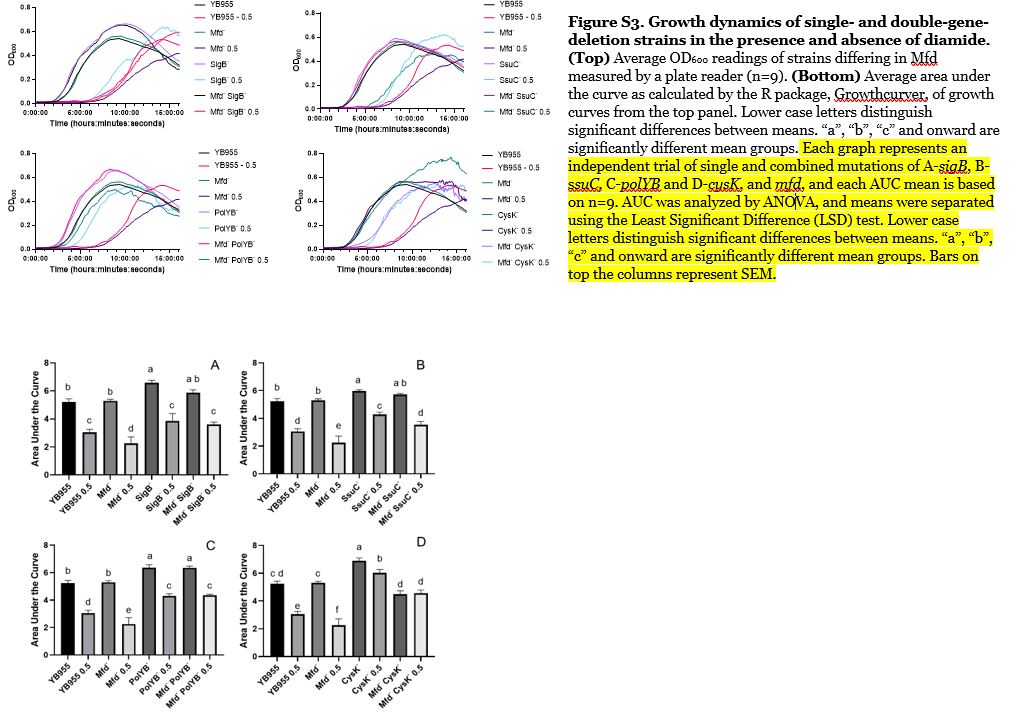

Supplement: Supplementary file 3 [file Image_3.jpg]

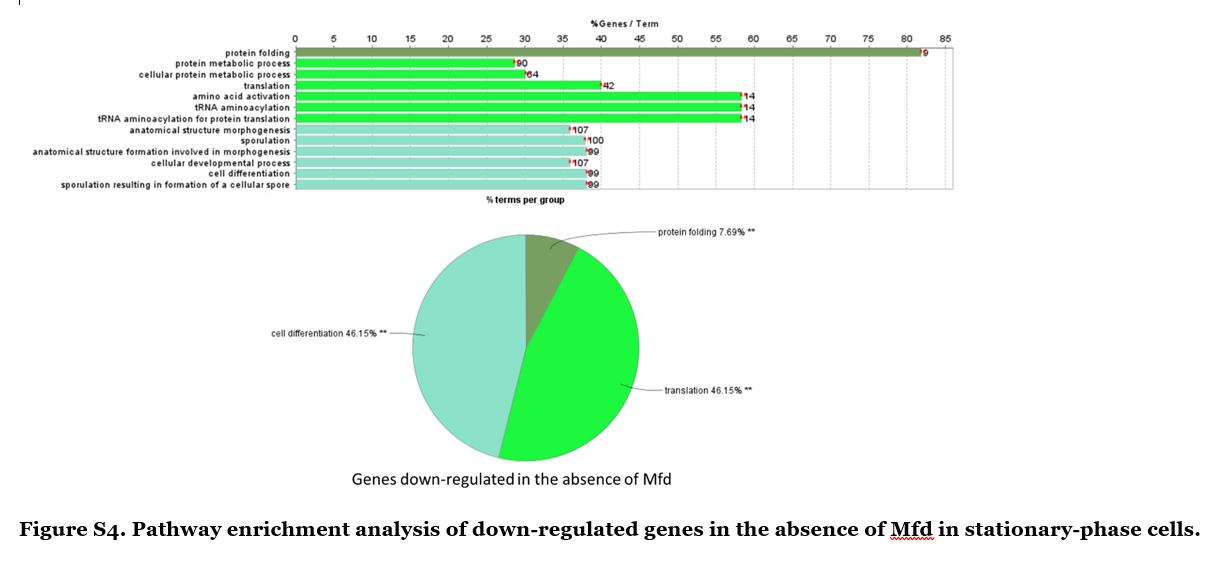

Supplement: Supplementary file 4 [file Image_4.JPEG]

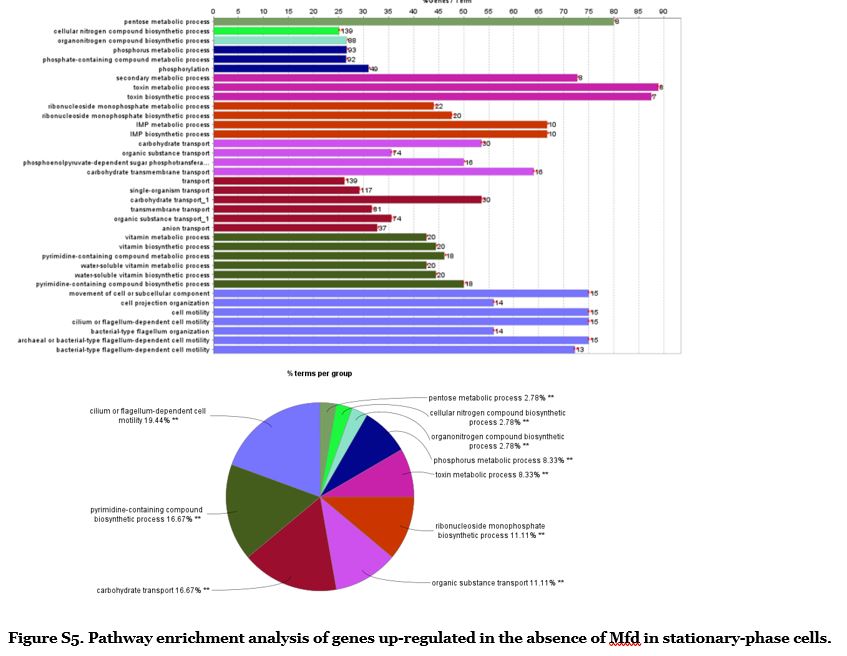

Supplement: Supplementary file 5 [file Image_5.JPEG]

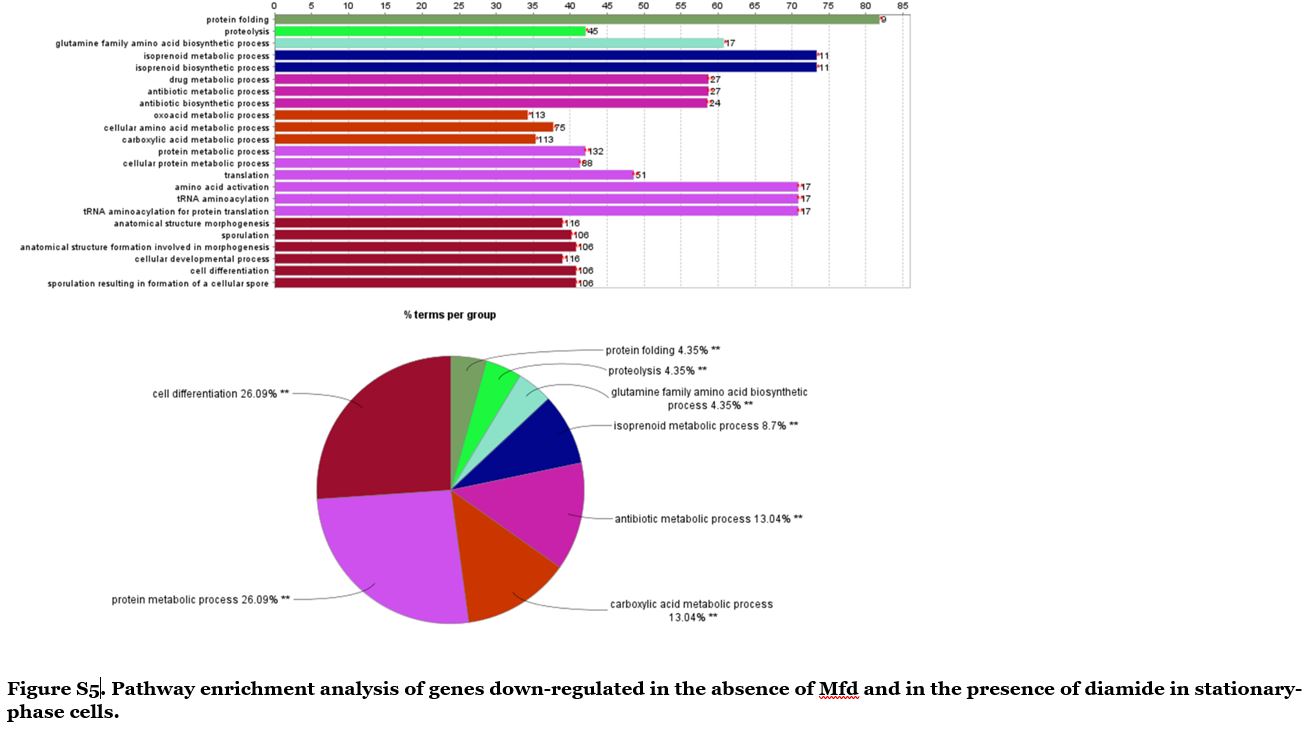

Supplement: Supplementary file 6 [file Image_6.JPEG]

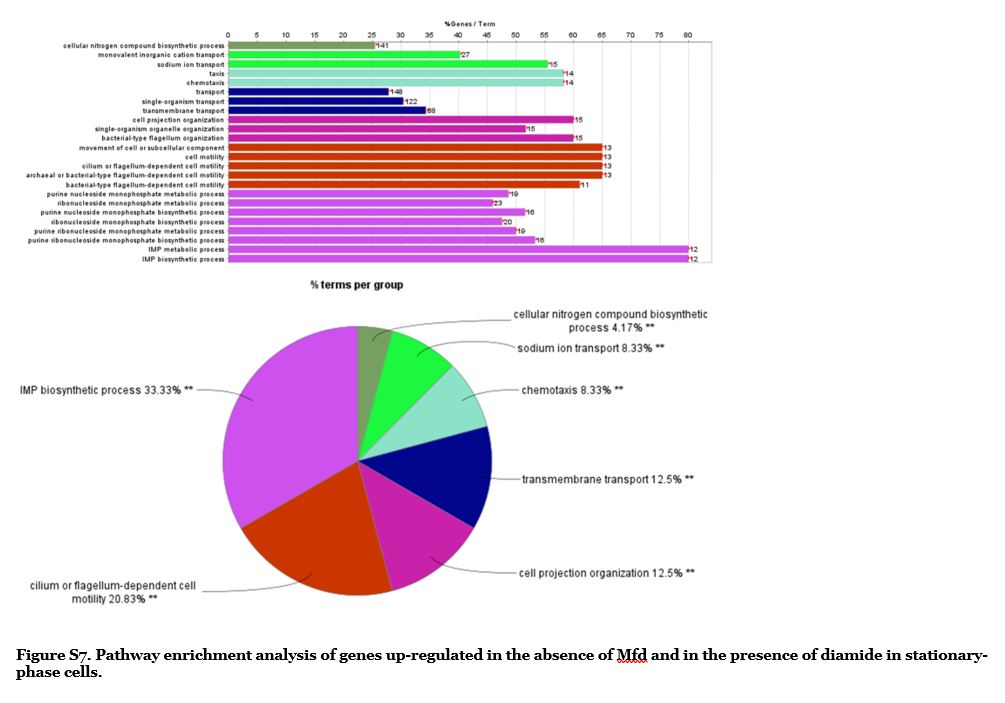

Supplement: Supplementary file 7 [file Image_7.JPEG]
