# Supplementary material for: Mfd Affects Global Transcription and the Physiology of Stressed Bacillus subtilis Cells
Source: Front Microbiol. 2021 Jan 28;12:625705. doi: 10.3389/fmicb.2021.625705 (PMC7885715; doi:10.3389/fmicb.2021.625705)
Supplement: Supplementary file 13 [file Table_6.DOCX]

**Supplementary Materials.**

**Table S6. Description of genes chosen to investigate Mfd genetic interaction and their effects on disulfide stress.**

| **Gene** | **log_2_FoldChange** | **function** | **Regulon** | **Oxidative/Electrophile Stimulon Member** |
| --- | --- | --- | --- | --- |
| *cysK* | -1.335166409 | biosynthesis of cysteine, control of CymR activity | CymR regulon, Spx regulon, SigA regulon, SigM regulon | no |
| *ssuC* | -1.090178022 | sulfonate uptake | CymR regulon, SigA regulon | no |
| *bstA* | -0.974382296 | detoxification; bacillithiol S-transferase | BstA regulon | yes |
| *ohrB* | -1.542418572 | organic peroxide resistance | SigB regulon | yes |
| *polYB (polY2)* | -1.149041837 | UV-targeted mutagenesis | LexA regulon | no |
| *aldY* | -1.356597831 | stress resistance; aldehyde dehydrogenase (NAD) | SigB regulon | yes |
| *cypC* | -1.37096633 | biosynthesis of beta-hydroxy fatty acid for lipopeptides | SigB regulon | yes |
| *ohrR* | n/a | regulation of ohrA expression in response to organic peroxides | SigA regulon, OhrR regulon | yes |
| *sigB* | n/a | general stress response, biocontrol of fungal growth | SigA regulon, SigB regulon, CcpA regulon | no |
| *perR* | n/a | regulation of the response to peroxide | PerR regulon, SigA regulon | yes |
| *yodB* | n/a | regulation of quinone and diamide detoxification | YodB regulon | yes |
| *sodA* | -1.06855698 | detoxification of oxygen radicals | SigB regulon | yes |
| *bshA* | -1.702028866 | biosynthesis of bacillithiol | Spx regulon, SigA regulon | yes |
| *bshB1* | -1.493706871 | biosynthesis of bacillithiol | Spx regulon, SigA regulon | yes |
| *ykuV* | -1.204354124 | protection of proteins against oxidative damage | AbrA regulon | yes |
